# Editorial: Advances in detection and control of post-harvest pathogens

**DOI:** 10.3389/fmicb.2023.1184039

**Published:** 2023-03-28

**Authors:** Khamis Youssef, Sergio Ruffo Roberto, Antonio Ippolito, Kamal A. M. Abo-Elyousr

**Affiliations:** ^1^Agricultural Research Center, Plant Pathology Research Institute, Giza, Egypt; ^2^Agricultural and Food Research Council, Academy of Scientific Research and Technology (ASRT), Cairo, Egypt; ^3^Agricultural Research Center, State University of Londrina, Londrina, PR, Brazil; ^4^Department of Soil, Plant and Food Science, University of Bari “Aldo Moro”, Bari, Italy; ^5^Department of Arid Land Agriculture, Faculty of Meteorology, Environment and Arid Land Agriculture, King Abdulaziz University, Jeddah, Saudi Arabia; ^6^Department of Plant Pathology, Faculty of Agriculture, University of Assiut, Assiut, Egypt

**Keywords:** foodborne pathogens, fungal pathogens, bacterial pathogens, post-harvest disease, detection, contamination

## Introduction

Post-harvest pathogens can cause disease in harvested products during transportation, handling, packaging, and storage. Such pathogens can cause serious damage in the fresh produce supply chain, with post-harvest losses of fresh fruit and vegetables estimated to be up to 50%. Rotting is a major issue in such losses, being caused mainly by fungal pathogens after fruit ripening (Junior et al., 2019). For fresh produce, heat-based treatments are difficult to apply during the post-harvest packing process. Current processes usually rely on washing, which can enhance contamination or leave the produce vulnerable to contamination. Inadequate storage of fresh produce may also provide ideal conditions for pathogens to grow. However, advances in detection and control methods have greatly improved our ability to manage these pathogens (Teixeira et al., 2021). Further research is needed to develop more sustainable and environmentally friendly methods for controlling these pathogens (Salem et al., 2016). Traditional testing methods for post-harvest pathogens can take days to produce results, which can lead to significant losses. However, rapid testing methods such as PCR and ELISA can detect pathogens in just a few hours, allowing for quicker identification and control (Law et al., 2015).

The aim of this Research Topic was to further understanding of bacteria and fungi causing post-harvest disease, and present future directions to improve the detection and control of such pathogens. Thirteen articles were accepted for this Research Topic dealing with grapes, blueberries, apples, Jerusalem artichoke, wolfberries, Shengzhou nane, and goji fruit. In this context, Ji et al. investigated the effects of ethanol vapor on the inhibition of *Alternaria alternata* and *Botrytis cinerea* in post-harvest blueberry and the induction of defense-related enzymes (DREs) activities in fungi-inoculated blueberries stored at 0 ± 0.5°C for 16 days. The authors found that ethanol vapor markedly inhibited the mycelial growth of all pathogens. Also, ethanol vapor enhanced the activities of DREs in fungi-inoculated blueberries, including β-1,3-glucanase (GLU), chitinase (CHI), phenylalnine ammonialyase (PAL), peroxidase (POD), and polyphenol oxidase (PPO). The findings of this study suggest that ethanol could be used as an activator of defense responses in blueberry against Alternaria and Botrytis rots, by activating DREs, having practical application value in the preservation of quality of fruits and vegetables during post-harvest phase. Guan et al. in their review discussed the regulatory requirements on gaseous interventions as well as organic production and handling of apples that could contribute to future industrial application and benefit the apple processors. Maluleke et al. tested 31 yeast strains from 21 species using different agar media and liquid mixed cultures. *Pichia kudriavzevii* was the most potent against *B. cinerea*, but with a narrow activity spectrum. Twelve other strains had broad antagonistic activity against multiple fungi. Most antagonistic strains produced chitinases and glucanases when exposed to *B. cinerea*. Analysis of volatile and non-volatile compounds produced by antagonistic yeast strains in the presence of *B. cinerea* revealed higher amount of alcohols, esters, organosulfur compounds, monoterpenes, cyclic peptides, and diketopiperazine. This study is the first to demonstrate the inhibitory effect of non-volatile compounds produced by various yeast species. Ling et al. investigated the inhibitory effect of *Bacillus velezensis* L1 on post-harvest disease of wolfberry. *B. velezensis* L1 and its volatiles (2,3-butanedione) effectively reduced the decay caused by pathogenic fungi and prolonged storage time. These results provide favorable evidence for the biocontrol activity of strain L1 against *Alternaria iridiaustralis* and other important fungal pathogens, and also provide insights to develop storage systems for fresh fruits and vegetables after harvest. Charlermroj et al. investigated a new lateral flow test strip assay that uses phage from microarray screening to distinguish live from dead *Salmonella enteritidi*s cells. The assay only takes 15 min, much shorter than culture-based methods that typically need 24 h. This screening method can be adapted for phage-based binder selection against other pathogen targets, eliminating the need for animal immunization. The phage-based lateral flow test strip assay is not limited to foodborne pathogens and could be used to test other targets, such as viruses. Guo T.-R. et al. isolated and identified *Aspergillus niger* as the pathogen causing fruit rot in Shengzhou nane fruit. Optimal growth conditions were determined, and a combined treatment of sodium bicarbonate (SBC) and natamycin (NT) showed high antifungal efficacy against *A. niger*, damaging cell and mitochondrial membrane integrity and disrupting energy metabolism. *In vivo* experiments showed a reduction in rot lesion diameter and decay rate in Shengzhou nane fruit with the combined treatment of SBC and NT. The study suggests that this combined treatment could be a viable alternative to synthetic fungicides for controlling post-harvest fruit decay caused by *A. niger*. Du et al. conducted a study on Jerusalem artichoke to determine the dynamics of the microbiome during storage in relation to varieties and various other factors. They found that *Flavobacterium, Sphingobacterium, Staphylococcus, Dysgonomonas, Acinetobacter*, and *Serratia* played important roles. The bacterial community was affected by crop genotype and carbohydrate structure. Wang et al. found that the main pathogens causing post-harvest decay of bagging-free apples were *Botryosphaeria dothidea* and *Colletotrichum gloeosporioides*, and that cinnamon and clove essential oils (EOs) were effective in limiting fungal growth and reducing rot in both *in vitro* and *in vivo* trials. The study also noted that the two identified organisms had different levels of resistance to the tested EOs, and that further research would be conducted on the synergistic effects of cinnamon and clove EOs for controlling post-harvest spoilage in bagging-free apples. Zhao et al. conducted a study on the effect of carvacrol (CVR) on *Alternaria alternata* demonstrating its damaging effect on pathogen by altering the integrity of the mycelial cell wall. Meanwhile, both the contents of cell wall polycarbohydrates containing chitin and β-1,3-glucan and the activities of the enzymes related to the biosynthesis of these polycarbohydrates were significantly decreased by CVR treatment, while the activities of chitinase and β-1,3-glucanase response of degrading the two polycarbohydrates were increased by CVR treatment. Sun et al. suggested that *Pseudomonas syringae* strain B-1 may be a promising candidate for the biological control of post-harvest apple ring rot, potentially reducing the need for chemical fungicides and improving the sustainability of apple production. Also, Esteves et al. confirmed the potential of wine yeasts as biocontrol agents, while highlighting the need for the establishment of fit-for-purpose selection programs depending on the mold target, the timing, and the mode of application. Ou et al. concluded that formation of the viable but non-culturable (VBNC) state in methicillin-resistant *Staphylococcus aureus* (MRSA) strains was verified, then two propidium monoazide-crossing priming amplification assays were developed and applied to detect MRSA in the VBNC state from pure culture and food samples.

## Author contributions

KY, SR, AI, and KA-E: writing—review and editing. All authors contributed to the article and approved the submitted version.
